# Hepatitis B virus replication and sex-determining region Y box 4 production are tightly controlled by a novel positive feedback mechanism

**DOI:** 10.1038/srep10066

**Published:** 2015-05-13

**Authors:** Jian Shang, Yuan Zheng, Xiaohong Guo, Jiayin Mo, Xueping Xie, Ying Xiong, Yingle Liu, Kailang Wu, Jianguo Wu

**Affiliations:** 1 State Key Laboratory of Virology and College of Life Sciences, Wuhan University, Wuhan 430072, P.R. China

## Abstract

Hepatitis B virus (HBV) infection is a major cause of liver diseases. However, the mechanisms underlying HBV infection and pathogenesis remain largely unknown. The sex-determining region Y box 4 (Sox4) is a transcriptional factor, which preferentially regulates the development of various organs, tissues, and cancers. But, the role of Sox4 in viral infection and pathogenesis has not been elucidated. Here, we demonstrated that Sox4 is up-regulated by HBV, and revealed the mechanism by which HBV regulates Sox4 expression. First, HBV stimulates Sox4 expression through transcriptional factor Yin Yang 1 (YY1), which binds to Sox4 promoter to activate Sox4 transcriptional activity. Second, miR-335, miR-129-2 and miR-203 inhibit Sox4 expression by targeting its mRNA 3’UTR, while HBV suppresses the microRNAs expression, resulting in up-regulating Sox4 post-transcriptionally. Third, Sox4 protein is degraded by proteasome, while HBV surface protein (HBsAg) prevents Sox4 from degradation by directly interacting with the protein, thereby enhancing Sox4 production post-translationlly. More interestingly, HBV-activated Sox4 in turn facilitates HBV replication by direct binding to the viral genome via its HMG box. Thus, this study revealed a novel positive feedback mechanism by which Sox4 production and HBV replication are tightly correlated.

Hepatitis B virus (HBV) infection causes major public health problems with an estimated 400 million people are chronic HBV carriers, many of them suffer from progressive forms of liver disease and are at high risk of developing hepatocellular carcinoma (HCC)^1^,^2^,^3^,^4^,^5^. HBV contains a partially double-stranded relaxed circular DNA (rcDNA) of 3200 nucleotides (nt) consisting of four partially overlapping open-reading frames (ORFs) that encode seven proteins: surface proteins (PreS1, PreS2 and HBsAg), core protein (HBcAg), e antigen (HBeAg), polymerase (HBp), and X protein (HBx)^6^,^7^. HBV core-associated DNA and pregenomic RNA (pgRNA) indicate persistent viral replication^8^. HBV infection stimulates a series of signaling pathways and regulates cell cycle and proliferation^9^, while host factors and pathways determining viral replication fitness contribute to the development of viral-associated diseases^10^,^11^,^12^. However, the mechanisms underlying HBV infection and pathogenesis remain largely unknown.

To elucidate the mechanism underlying HBV infection and pathogenesis, we initially performed cDNA microarrays, which revealed that the sex-determining region Y box 4 (Sox4) expression is closely related to HBV infection and HCC formation (Table S1 and S2). Sox4, a transcription factor in the sex-determining region Y (SRY) family, contains a high-mobility group (HMG) box DNA-binding domain (DBD) at N-terminus and a transactivation domain (TAD) at C-terminus. It binds preferentially to AACAAAG motif in the minor groove of targeted genes^13^,^14^. Sox4 is involved in the development of various organs and tissues, including heart^15^, nervous system^16^, osteoblasts^17^, and endocrine islets^18^. Sox4 leads to tumor formation by suppressing p53-induced BCL2-associated X protein (Bax) expression and inducing epithelial-mesenchymal transition^19^,^20^. It also contributes to breast cancer progression and regulates melanoma cell migration and invasion^21^. Sox4 expression is induced by transforming growth factor-beta (TGF-β) and subsequently suppresses GATA binding protein-3 (GATA-3)^22^. Conversely, Sox4 expression is inhibited by miR-129-2 and miR-335-5p, which target the 3’ UTR of its mRNA^23^,^24^,^25^. However, the role of Sox4 in viral infection has not been reported. In this study, we evaluated the correlation between HBV replication and Sox4 expression with the aim of elucidating the mechanism underlying HBV infection and Sox4 function, which would provide new insights into HBV infection and pathogenesis.

## Results

### Sox4 expression is elevated in HBV-associated HCC tissues and HBV-transfected cells

**Figure 1:**
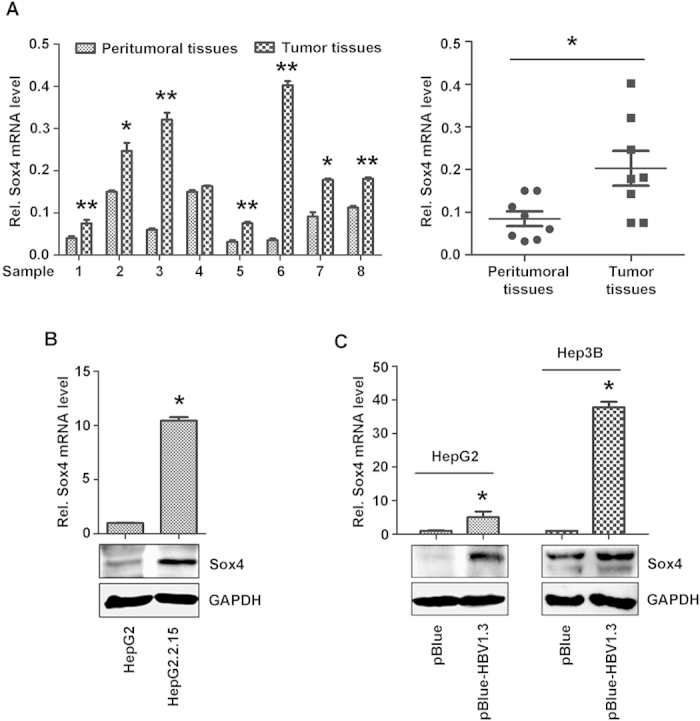
Sox4 expression is elevated in HBV-associated HCC tissues and HBV-transfected cells. (**A**) Total RNAs were extracted from 8 pairs of HBV-associated HCC tissues and adjacent liver tissues using TRIzol reagent, and then reverse-transcribed. Sox4 mRNAs were analyzed using real-time PCR. (**B**) Sox4 mRNA (upper panel) and protein (lower panel) were detected in HepG2.2.15 compared with HepG2 cells by real-time PCR and Western blotting. (**C**) HepG2 and Hep3B cells were transfected with pBlue-HBV1.3 or pBlue. Sox4 mRNA (upper panel) and protein (lower panel) level were detected in HBV-transfected cells compared with pBlue-transfected cells by real-time PCR and Western blotting. *P < 0.05.

The correlation between HBV infection and Sox4 expression was initially evaluated by three approaches. First, Sox4 mRNAs in 8 pairs of clinical samples were measured and compared. Sox4 mRNAs were much higher in HBV-associated HCC tissues compared with the corresponding peritumoral tissues (Fig. 1A, left panel); and the average mRNA level of Sox4 was clearly higher in HBV-related HCC tissues than that in peritumoral tissues (Fig. 1A, right panel). Next, Sox4 expression levels in cultured cells were evaluated. Sox4 mRNA and protein levels were significantly higher in HepG2.2.15 cells compared with HepG2 cells (Fig. 1B). Finally, Sox4 expression levels in HBV-transfected cells were analyzed. Sox4 mRNA and protein were higher in HBV-transfected cells compared with mock-transfected cells (Fig. 1C). Thus, we revealed that Sox4 expression is highly associated with HBV replication.

### HBV activates Sox4 transcriptionally by inducing YY1 expression through the MAPK signaling

**Figure 2:**
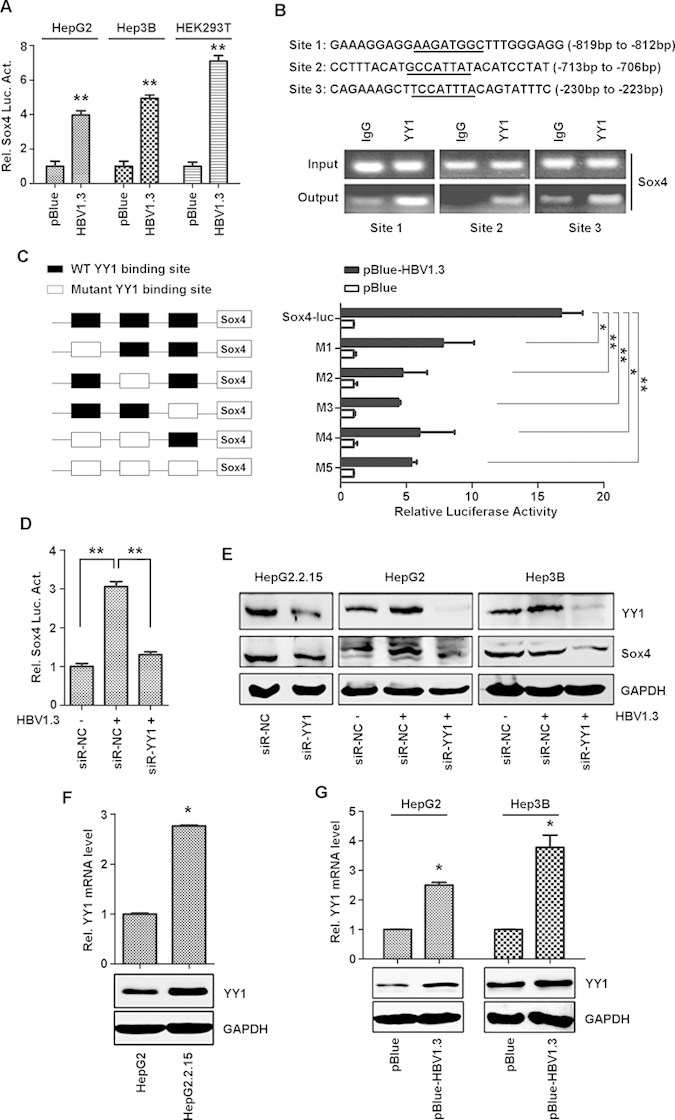
Transcription factor YY1 is required for HBV-activated Sox4 expression. (**A**) Sox4 promoter activity was detected in three cell lines co-transfected with pBlue-HBV1.3 or pBlue and pSox4-Luc. Luciferase activity was measured using a TD-20/20 luminometer and normalized to the control. The results are presented as means ± SD (*n* = 3). (**B**) The potential YY1 binding sites on Sox4 promoter were shown (upper panel). ChIP assays demonstrate that YY1 binds to the three potential binding sites on Sox4 promoter (lower panel). (**C**) Sox4 promoter containing three YY1 binding sites and its mutant promoters carrying deleted YY1 sites were generated and sub-cloned into pGL3-Basic to create pSox4-Luc and its mutant pSox4-Luc (M1-M5) (left panel). Cells were co-transfected with the indicated plasmids. Luciferase assays were performed and normalized to the control (right panel). The results were presented as means ±SD (*n* = 3). (**D**) HepG2 cells were co-transfected with indicated plasmids and siRNA mimics. Luciferase activity was measured and normalized to the control. The results were presented as means ±SD (*n* = 3). (**E**) HepG2.2.15 cells were transfected with siR-NC or siR-YY1 mimics, while HepG2 and Hep3B cells were co-transfected with pBlue or pBlue-HBV1.3 and siR-YY1 or siR-NC. The indicated proteins were analyzed by Western blotting. (**F and G**) YY1 mRNA (upper panel) and protein (lower panel) levels were detected in HepG2.2.15 compared with HepG2 cells or in HBV-transfected cells compared with pBlue-transfected cells. *P < 0.05, **P < 0.01.

The mechanism by which HBV regulates Sox4 expression was then elucidated. Cells were co-transfected with pBlue-HBV1.3, which contains a 1.3-fold length of HBV genome as described previously^26^ and pSox4-Luc, in which luciferase is under the control of Sox4 promoter. The promoter activity was up-regulated significantly in pBlue-HBV1.3-transfected cells (Fig. 2A), suggesting that HBV stimulates Sox4 expression by regulating its promoter. Sequence analyses of Sox4 promoter revealed that it contains 3 potential binding elements for the transcription factor Yin Yang 1 (YY1) (Fig. 2B, upper panel). YY1 regulates many genes associated with cell growth and differentiation by binding to a consensus DNA sequence GCCATNTT/AANATGGC of targeted genes^27^,^28^. Chromatin Immunoprecipitation (ChIP) assays showed that YY1 could bind to the 3 potential binding elements in Sox4 promoter (Fig. 2B, lower panel), suggesting that YY1 is a key factor in HBV regulating Sox4 expression by stimulating its promoter activity.

The involvement of YY1 in HBV-activated Sox4 expression was confirmed using two approaches. First, 5 mutants (M1-M5) of YY1 binding elements in Sox4 promoter were introduced into pSox4-Luc to generate 5 mutant reporters (Fig. 2C, left panel). HepG2 cells were co-transfected with pBlue-HBV1.3 and pSox4-Luc or the mutants. The activities of Sox4 mutant promoters were much lower compared with the wild-type promoter (Fig. 2C, right panel), suggesting that YY1 binding sequences are required for HBV-activated Sox4 expression. Next, HepG2 cells were co-transfected with pBlue-HBV1.3 and a siRNA specific to YY1 (siR-YY1) or its control siRNA (siR-NC). Sox4 promoter activity was up-regulated in the presence of siR-NC and down-regulated in the presence of siR-YY1 in HBV-transfected cells (Fig. 2D). In addition, Sox4 protein was also repressed by siR-YY1 in HepG2.2.15 cells and in pHBV1.3-transfected HepG2 and Hep3B cells (Fig. 2E). These results demonstrated that knock-down of YY1 down-regulates HBV-activated Sox4 expression, and that YY1 plays a critical role in HBV-activated Sox4 expression.

The role of HBV in the regulation of YY1 expression was also investigated. Results showed that YY1 mRNA and protein were higher in HepG2.2.15 cells than in HepG2 cells (Fig. 2F), and up-regulated in HBV1.3-transfected HepG2 and Hep3B cells compared with control cells (Fig. 2G), suggesting that HBV activates YY1 expression. The signaling components involved in HBV-regulated YY1 expression were then determined. YY1 expression was significantly reduced by inhibitors of the MAPK signaling components, SP600125 (JNK inhibitor), GF109203 (protein kinase C inhibitor), and U0126 (ERK inhibitor) (Fig. S1A), indicating that HBV enhances YY1 expression through activating the MAPK signaling.

Because both Sox4 and YY1 are regulated by HBV, the temporal expression patterns of Sox4 and YY1 mediated by HBV were evaluated. In HBV-transfected HepG2 cells, YY1 protein was activated at 12 h post-transfection, whereas Sox4 protein was stimulated at 24 h post-transfection and in HBV-transfected Hep3B cells, YY1 expression was up-regulated at 12 h post-transfection, while Sox-4 expression was increased at 36 h post-transfection (Fig. S1B). These results suggested that HBV activates the MAPK pathway to induce YY1 expression, which then binds to Sox4 promoter to activate its transcription.

### HBV up-regulates Sox4 post-transcriptionally by inhibiting miR-335, miR-129-2, and miR-203 through epigenetic modification

**Figure 3:**
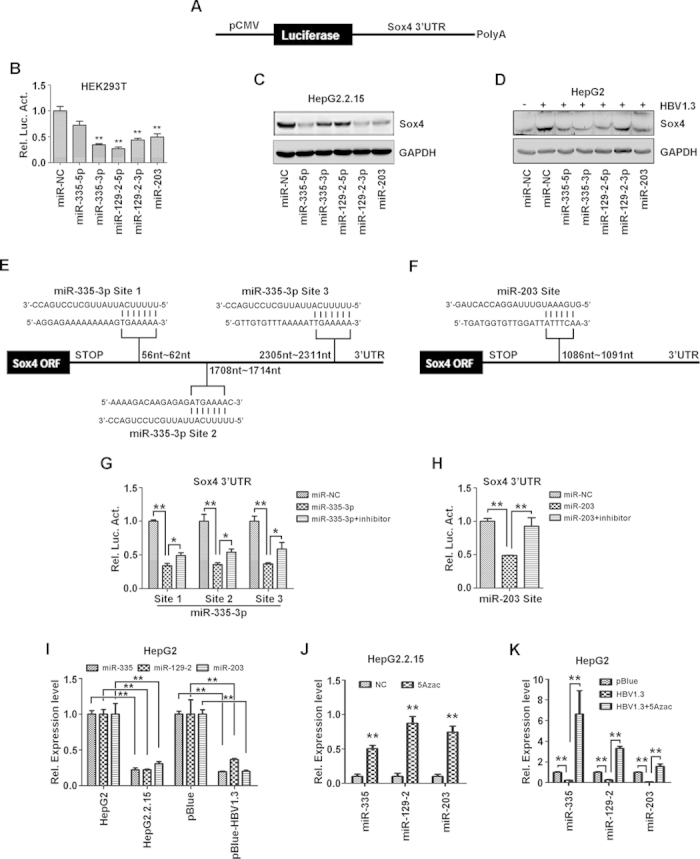
HBV up-regulates Sox4 post-transcriptionally by inhibiting miR-335, miR-129-2, and miR-203 through epigenetic modification. (**A**) Diagram of pSox4 3’UTR-Luc. The full-length 3`-UTR of Sox4 was sub-cloned into pCMV-Luc. (**B**) HEK293T cells were co-transfected with pSox4 3`UTR-Luc and indicated miRNA mimics. Luciferase activity was measured and normalized to the control. The results are presented as means ±SD (*n* = 3). (**C**) HepG2.2.15 cells were transfected with the miRNA mimics. (**D**) HepG2 cells were co-transfected with pBlue-HBV1.3 and the miRNA mimics. (**C and D**) SOX4 expression was analyzed by Western blotting. (**E and F**) Diagram of three predicted target sites for miR-335-3p and one predicted target site for miR-203 in Sox4 3’UTR. Each site was constructed to pCMV-Luc separately. (**G and H**) Cells were co-transfected with the indicated Sox4 3’UTR plasmids and miRNA or miRNA inhibitor. Luciferase activity was measured and normalized to the control. The results are presented as means ± SD (*n* = 3). (**I**) HepG2 cells were tra*n*sfected with pBlue or pBlue-HBV1.3. (**J**) HepG2.2.15 cells were treated with 5-azacytidine (5`-Azac), an inhibitor of DNA methyltransferase, or the control (NC). (**K**) HepG2 cells were transfected with pBlue or pBlue-HBV1.3, and treated with 5`-Azac or NC. (I-K) Total RNAs were extracted from transfected cells and reverse-transcribed. The miRNAs were determined by real-time PCR. *P < 0.05, **P < 0.01.

Previous studies reported that miR-335 and miR-129 inhibit Sox4 expression by targeting the 3’ UTR of Sox4 mRNA, and that miR-203 potentially regulates Sox4 expression^25^,^29^. Therefore, we assessed whether miR-335-5p, miR-129-2, and miR-203 are involved in HBV-regulated Sox4 expression. A reporter plasmid pCMV-Luc-Sox4 3’UTR, containing cytomegalovirus (CMV) promoter, luciferase (Luc), and the full length of Sox-4 3’UTR, was constructed (Fig. 3A). Sox4 3’UTR activity was reduced by miR-335-3p, miR-129-2-5p, miR-129-2-3p, and miR-203 mimics in transfected HEK293T cells (Fig. 3B). Sox4 protein expression was down-regulated by miR-335-5p, miR-129-2-3p, and miR-203 mimics in HepG2.2.15 cells (Fig. 3C). Moreover, HBV-induced Sox4 protein was down-regulated by miR-335-5p, miR-335-3p, miR-129-2-5p, miR-129-2-3p, and miR-203 mimics in HepG2 cells (Fig. 3D). These results indicated that miR-335, miR-129-2, and miR-203 down-regulate HBV-activated Sox4 expression.

Sequencing analysis revealed that Sox4 3’-UTR contains three putative binding sequences for miR-335-3p (Fig. 3E), and one for miR-203 (Fig. 3F). According to the sequences, four luciferase reporters of Sox4 3’UTR were constructed, which contain miR-335-3p site I, II and III, and miR-203 site, respectively. HepG2 cells were co-transfected with each of the reporters, miRNA mimics, and/or inhibitor. Luciferase assays showed that luciferase activities were down-regulated by miR-335-3p and miR-203, but such repressions were prevented by the inhibitors (Fig. 3G,H), demonstrating that miR-335, miR-129-2, and miR-203 down-regulate Sox4 expression by direct targeting its 3’UTR.

Since Sox4 expression was reduced by miR-335, miR-129-2, and miR-203, the effects of HBV on the expression of miR-335, miR-129-2, and miR-203 were evaluated. The levels of miR-335, miR-129-2, and miR-203 were down-regulated significantly in HepG2.2.15 cells compared with HepG2 cells, and in HBV-transfected HepG2 cells compared with the negative control cells (Fig. 3I), indicating that HBV represses miR-335, miR-129-2, and miR-203 expression. The promoters of miR-335, miR-129-2, and miR-203 contain CpG islands that are usually methylated and suppressed in many tumor formation conditions^30^,^31^. Thus, we determined whether HBV down-regulates the miRNAs expression *via* epigenetic modification or methylation. In HepG2.2.15 cells, miR-335, miR-129-2, and miR-203 were inhibited, but up-regulated significantly by the DNA methyltransferase (DNMT) inhibitor, 5-azacytidine (5’-Azac) (Fig. 3J). In HepG2 cells, miR-335, miR-129-2, and miR-203 were repressed by transfected HBV, but activated significantly by 5’-Azac (Fig. 3K). These results suggested that methylation plays an important role in HBV-regulated expression of miR-335, miR-129-2, and miR-203, and that HBV activates Sox4 expression by inhibiting miR-335, miR-129-2, and miR-203 through epigenetic modification.

Because we revealed that HBV activates Sox4 expression by two mechanisms, activating YY1 expression and repressing miRNAs expression, we further investigated the correlation between the two events. In one hand, the effect of miRNAs on YY1 expression was evaluated. In HepG2.2.15 cells, YY1 mRNA was down-regulated by miR-335-5p, miR-335-3p, miR-129-2-5p, and miR-203 mimics (Fig. S2A). In Hep3B cells, YY1 mRNA expression was reduced by miR-335-5p, miR-335-3p, miR-129-2-5p, miR-129-2-3p, and miR-203 mimics (Fig. S2B). These results suggested that miR-335, miR-129-2, and miR-203 play inhibitory roles in YY1 expression. Sequencing analyses revealed that YY1 3’-UTR contains 2 potential target sites for miR-335, 4 for miR-129-2, and 2 for miR-203 (Fig. S2C). A reporter plasmid pYY1 3’UTR-Luc was constructed, in which luciferase is under the control of YY1 3’UTR, and then YY1 3’UTR activity was detected in the presence of miR-NC, but down-regulated significantly by miR-335-5p, miR-335-3p, miR-129-2-5p, miR-129-2-3p, and miR-203 mimics (Fig. S2D), indicating that these miRNAs can recognize YY1 3’-UTR RNA and thus to knock-down YY1 expression. On the other hand, the roles of YY1 in expression levels of miR-335, miR-129-2, and miR-203 were also determined. The levels of miR-335, miR-129-2, and miR-203 were significantly down-regulated by YY1 in HepG2 cells (Fig. S2E), suggesting that YY1 negatively regulates these miRNAs expression. Taken together, miR-335, miR-129-2, and miR-203 down-regulate YY1 expression, which subsequently represses the miRNAs expression. Thus, the two events, HBV promotes Sox4 expression by activating YY1 and by suppressing miR-335, miR-129-2, and miR-203, are independent but related.

### HBV up-regulates Sox4 post-translationally by protecting Sox4 from proteasome-mediated degradation through a direct interaction between HBs and Sox4

**Figure 4:**
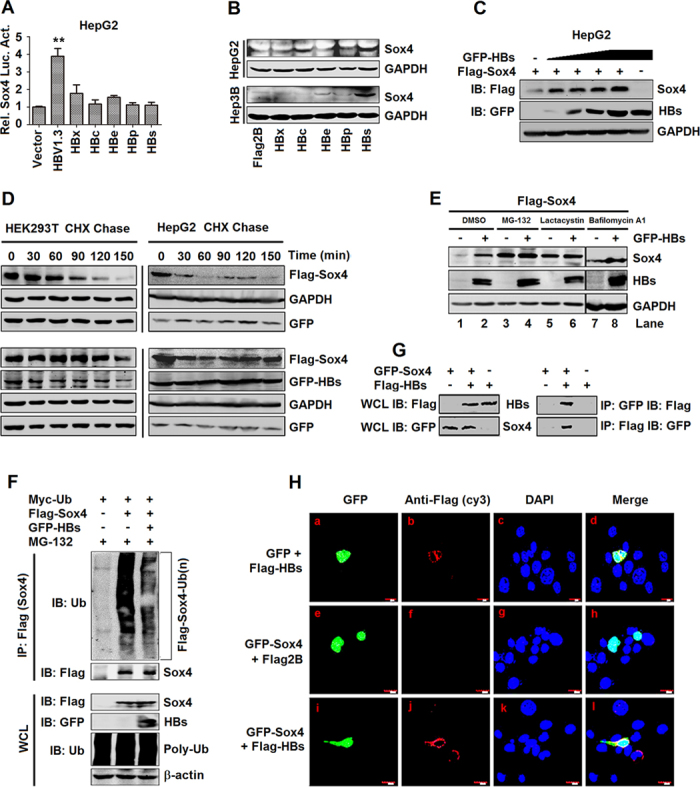
HBs protein inhibits ubiquitination-related degradation of Sox4 through direct interaction with the protein. (**A**) HepG2 cells were co-transfected with pSox4-Luc and plasmids expressing individual HBV protein, as indicated. Luciferase activity was measured and normalized to the control. The results are presented as means ± SD (*n* = 3). (**B**) HepG2 cells (upper panel) and Hep3B cells (lower panel) were transfected with plasmids expressing individual HBV proteins, as indicated. Sox4 expression was detected by Western blotting. (**C**) HepG2 cells were co-transfected with pFLAG-Sox4 and pGFP-HBsAg at different concentrations. Sox4 and HBsAg was detected by Western blotting. (**D**) HEK293T and HepG2 cells were co-transfected with pFLAG-Sox4 and pGFP, and treated with CHX (upper panels). Cells were co-transfected with pFLAG-Sox4 and pGFP-HBsAg, and treated with CHX (lower panels). The cells were lysed in lysis buffer containing protease inhibitors. The proteins were detected by Western blotting. (**E**) HEK293T cells were co-transfected with pFLAG-Sox4 and pGFP-HBsAg or pGFP, and treated with proteasome inhibitors (MG-132 or lactacystin) or lysosome inhibitor (Bafilomycin A1). Cells were lysed and the proteins were detected by Western blotting. (**F**) HEK293T cells were co-transfected with pFLAG-Sox4 or pFLAG and pGFP-HBsAg or pGFP and pMyc-Ub, and then treated with MG-132. Sox4-Ub(n) was performed by immunoprecipitation (upper panel) and analyzed by Western blotting. Whole cell lysates (WCL) were collected for the detection of each transfected plasmid (lower panel). (**G**) HEK293T cells were co-transfected with pFLAG-Sox4 or pFLAG and pGFP-HBsAg or pGFP. Co-immunoprecipitation was performed and immunoprecipitates were detected by Western blotting. (**H**) HepG2 cells were co-transfected with pFLAG-HBsAg and pGFP (a-d), with pGFP-Sox4 and pFLAG (**e-h**), or with pFLAG-HBsAg and pGFP-Sox4 (**i-l**). The locations of the indicated proteins were analyzed by immunofluorescence staining.

Because HBV activates Sox4 expression, we evaluated the roles of individual viral protein in the regulation of Sox4 expression. Sox4 promoter was stimulated by HBV, but not by the viral proteins, HBx, HBcAg, HBeAg, HBp, and HBsAg (Fig. 4A), suggesting that the viral proteins are not involved in regulating Sox4 at transcriptional level. However, Sox4 protein was up-regulated by HBs but not by the other proteins in HepG2 and Hep3B cells (Fig. 4B), indicating that HBs plays a role in activating Sox4 at the translational or post-translational level. Since the half-life of Sox4 protein is reportedly less than 1 h due to its degradation by the proteasome^32^, we speculated that the inconsistency of HBsAg-regulated Sox4 expression between the transcriptional and translational levels may result from the regulation of Sox4 degradation. To verify this speculation, firstly, Sox4 and HBs were over-expressed in 293T cells, Sox4 level was increased by HBsAg in a dose-dependent manner (Fig. 4C), suggesting that HBs enhances Sox4 protein stability. Secondly, Sox4 protein level was quickly reduced in the presence of a protein biosynthesis inhibitor, cycloheximide (CHX), in a time-dependent manner (Fig. 4D, upper panels), indicating that Sox4 has a short half-life time. However, such degradation was prevented by HBs (Fig. 4D, lower panels), demonstrating that HBsAg protects Sox4 protein from degradation to extend its half-life.

Two main protein degradation pathways exist in the cells, the lysosome pathway and the proteasome pathway^33^. We revealed that Sox4 level was higher in the presence of HBs than in the absence of HBs (Fig. 4E, lane 2 *vs*. 1), confirming that HBs enhances Sox4 level. In addition, in the presence of proteasome inhibitors, MG-132 (Fig. 4E, lane 3 and 4) and lactacystin (Fig. 4E, lane 5 and 6), Sox4 was not degraded, and not affected by HBsAg (Fig. 4E, lane 4 *vs*. 3 and lane 6 *vs*. 5). However, in the presence of a lysosome inhibitor, Bafilomycin A1, Sox4 was still degraded but enhanced by HBsAg (Fig. 4E, lane 8 *vs*. 7). These results demonstrated that Sox4 degradation is blocked by MG-132 and lactacystin, and suggested that HBsAg protects Sox4 from proteasome-mediated degradation. Because the C-terminal domain of Sox4 regulates its polyubiquitin-independent proteasome degradation^32^, we investigated whether HBs inhibits the ubiquitination-related proteasome degradation of Sox4. Sox4 polyubiquitin formation was clearly detected in the absence of HBsAg, but reduced significantly in the presence of HBsAg (Fig. 4F), demonstrating that HBs inhibits Sox4 polyubiquitin formation to enhance Sox4 stability.

The inhibition of protein ubiquitination often requires the interaction between proteins. We speculated that HBsAg may interact with Sox4 to inhibit its degradation. Sox4 and HBsAg were co-immunoprecipitated in the cells co-transfected with pGFP-Sox4 and pFlag-HBs (Fig. 4G), suggesting that Sox4 and HBsAg are interacted with each other. Since HBsAg is normally located in the cytoplasm and Sox4 is usually distributed in the nucleus, we verified how the interaction between the two proteins occurred. In the absence of Sox4, HBsAg was mainly located in the cytoplasm but not in the nucleus (Fig. 4Ha-d); while in the absence of HBsAg, Sox4 was located predominantly in the nucleus (Fig. 4He-h). However, in the presence of HBsAg, a proportion of Sox4 was co-localized with HBs in the cytoplasm, although a proportion of Sox4 remained in the nucleus (Fig. 4Hi-l). In addition, Sox4 level was enhanced by HBsAg in a dose-dependent manner in the cytoplasm (Fig. S3A), while Sox4 was not affected by HBsAg in the nucleus (Fig. S3B). These results suggested that HBsAg interacts with Sox4 in the cytoplasm to protect it from ubiquitination-related degradation.

### Sox4 facilitates HBV replication in hepatoma cells

**Figure 5:**
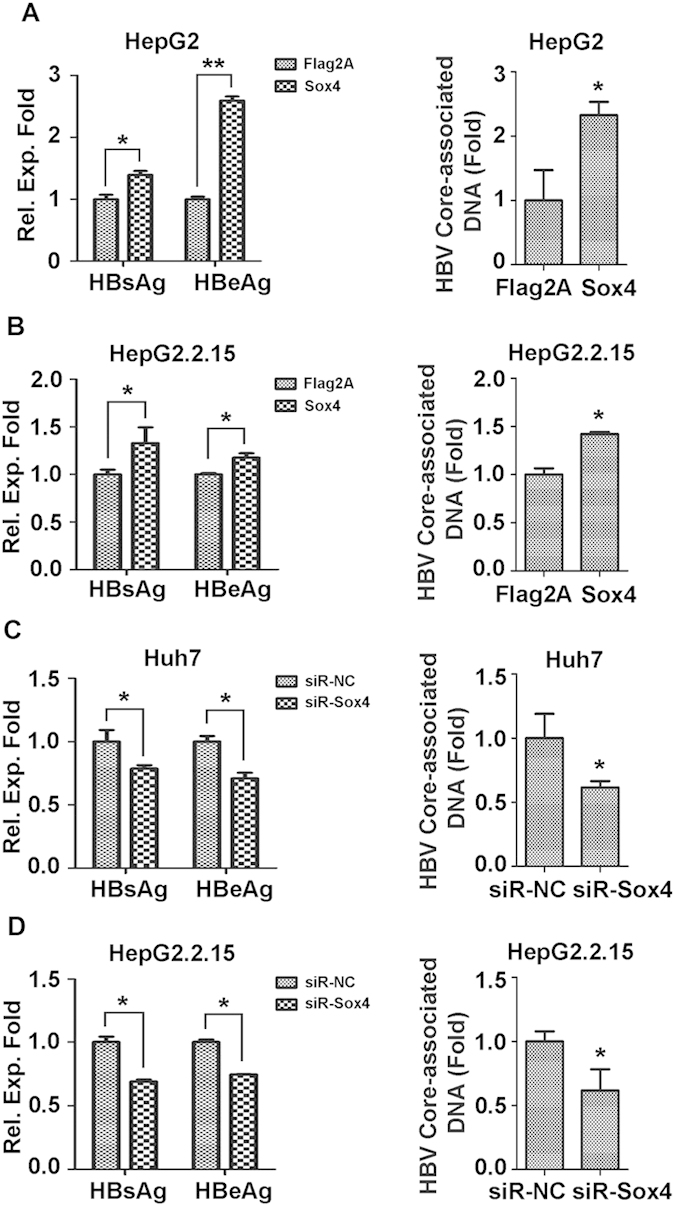
Sox4 facilitates HBV replication in hepatoma cells. (**A**) HepG2 cells were co-transfected with pFLAG-Sox4 or pFLAG2A and pBlue-HBV1.3 or pBlue. (**B**) HepG2.2.15 cells were transfected with pFLAG-Sox4 or pFLAG2A. (**C**) Huh7 cells were co-transfected with siR-Sox4 or siR-NC and pBlue-HBV1.3 or pBlue. (**D**) HepG2.2.15 cells were transfected with siR-Sox4 or siR-NC. (**A-D**) Cell were collected and centrifuged. Supernatants were analyzed using ELISA kits for HBs and HBe (left panels). Cells were treated with lysis buffer for HBV core-associated DNA extraction, and HBV core-associated DNA was analyzed by real-time PCR (right panels). *P < 0.05.

Because Sox4 expression is tightly controlled by HBV at multiple levels, we speculated that Sox4 may play an important role in HBV replication. The effect of Sox4 on HBV replication was evaluated by over-expression and knock-down of Sox4. HBs and HBe proteins expression (Fig. 5A) and HBV core-associated DNA (Fig. 5B) were significantly enhanced by Sox4 in HBV-transfected HepG2 cells. Similarly, HBs and HBe proteins (Fig. 5C) and HBV core-associated DNA (Fig. 5D) were up-regulated by Sox4 in HepG2.2.15 cells. These results demonstrated that over-expression of Sox4 results in the up-regulation of HBV replication.

To knock-down Sox4 expression, three small interfering RNA specific to Sox4 (siR-Sox4-1, 2, and 3) and a negative control siRNA (siR-NC) were synthesized. Sox4 protein was significantly reduced by siR-Sox4-1 and siR-Sox4-3, and down-regulated by siR-Sox4-2, but not affected by siR-NC (Fig. S4), indicating that siR-Sox4-1 and 3 are effective. HBs and HBe proteins expression (Fig. 5E) and HBV core-associated DNA replication (Fig. 5F) were down-regulated by siR-Sox4-3 compared with siR-NC in HBV-transfected Huh7 cells. Similarly, HBs and HBe proteins (Fig. 5G) and HBV core-associated DNA (Fig. 5H) were reduced by siR-Sox4-3 compared with siR-NC in HepG2.2.15 cells. These results indicated that knock-down of Sox4 leads to the down-regulation of HBV replication. Thus, we revealed that Sox4 facilitates HBV replication in hepatoma cells.

### Sox4 activates HBV replication by directly binding to the viral genome through its HMG domain

**Figure 6:**
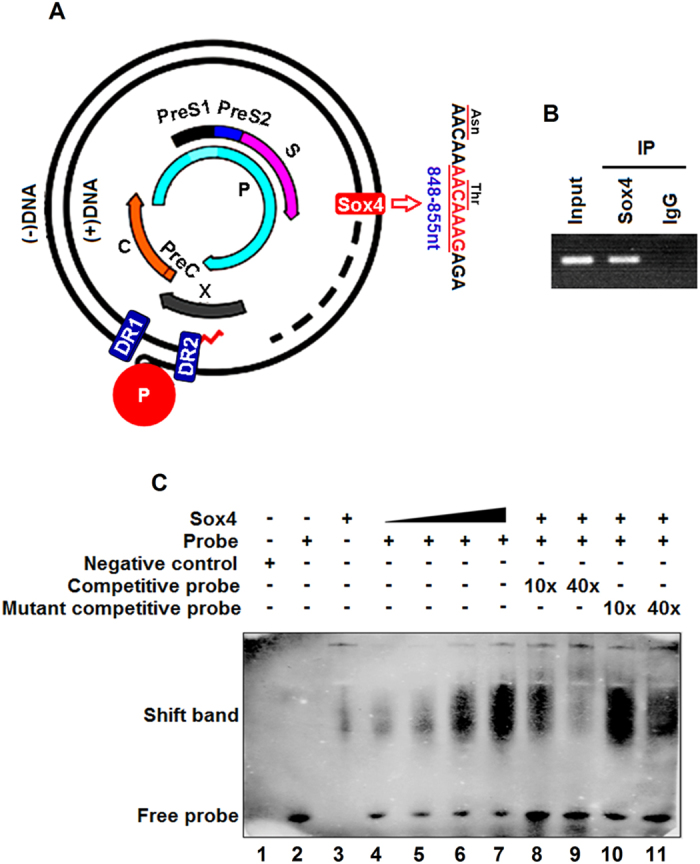
Sox4 binds directly to HBV genomic DNA. (**A**) Diagram of wild-type HBV genome(wt) and the potential Sox4 binding site located at 848-855 nt of HBV polymerase are indicated. (**B**) HepG2.2.15 cells were transfected with pFLAG-Sox4, and ChIP assay was performed to detect that Sox4 binds to the predicated site. (**C**) Electrophoretic mobility shift assay (EMSA) was performed by incubating recombinant Sox4 protein and labeled probe. To ensure the specific binding of protein, the probes were chased with a 10- and 40-fold molar excess of cold wild-type and mutant oligonucleotides. Samples were electrophoresed on non-denaturing polyacrylamide gels.

Sox4 regulates the expression of many genes by targeting the DNA sequences of their promoters. We speculated that Sox4 may regulate HBV replication by binding to HBV DNA. Sequencing analyses predicted that HBV genome contains a putative Sox4 binding site (AACAAAG) at 848-855 nt (Fig. 6A). To determine the ability of Sox4 binding to HBV genomic DNA, Sox4-transfected HepG2.2.15 cells were used for ChIP assay. The results revealed that Sox4 could recognize and bind to HBV genomic DNA (Fig. 6B). Further, Sox4 was expressed and purified in prokaryotic expression system (*E. coli*, BL21). The purified recombinant Sox4 protein was identified by Western blot analysis using anti-His antibody (Fig. S5). EMSA results demonstrated that shifted band was not detected in the presence of the negative control, the probe, or Sox4 protein alone, respectively (Fig. 6C, lanes 1-3). In contrast, a shifted band was detected in the presence of both the probe and Sox4 protein, and the intensity of shifted band was enhanced as Sox4 concentration increased (Fig. 6C, lanes 4-7), decreased by the competitive probe in a dose-dependent manner (Fig. 6C, lanes 8-9), but not affected by the mutant competitive probe (Fig. 6C, lanes 10-11). These results demonstrated that Sox4 recognizes and binds to the putative Sox4 binding sequence of HBV genome.

**Figure 7:**
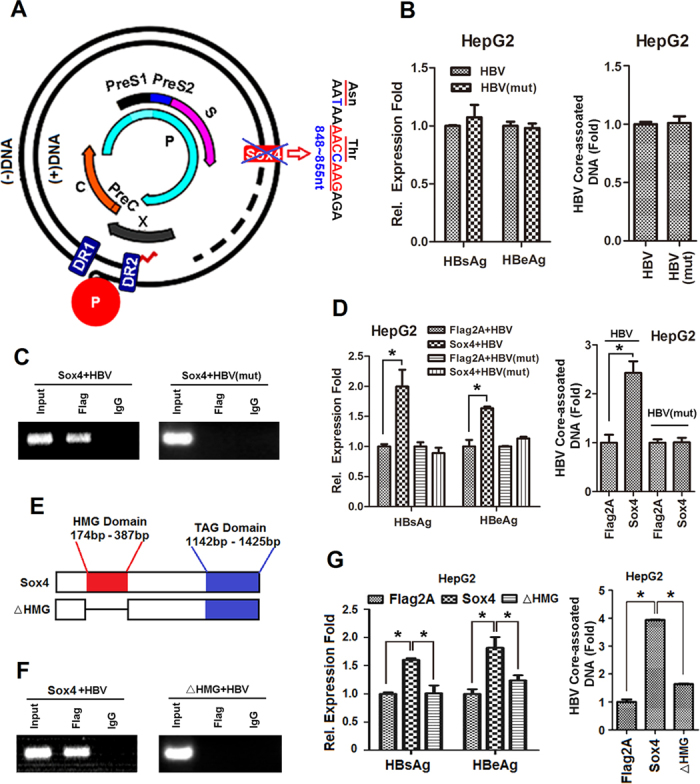
Sox4 activates HBV replication by binding to the viral genome through its HMG domain. (**A**) Diagram of mutant HBV genome(mut), in which two nucleotide mutations in Sox4 binding site of HBV genome(wt) were introduced without altering the amino acid sequences of HBV polymerase. (**B**) HepG2 cells were transfected with pHBV(wt) or pHBV(mut). HBsAg and HBeAg (left panel) were detected by ELISA, and HBV core-associated DNA was analyzed by qRT-PCR (right panel). (**C**) HepG2 cells were co-transfected with pFLAG-Sox4 and pHBV(wt) or pHBV(mut). ChIP assays were performed to detect that Sox4 can not bind to the mutant HBV. (**D**) HepG2 cells were co-transfected with pFLAG-Sox4 or pFLAG and pHBV(wt) or pHBV(mut). Cells were collected centrifuged. Supernatants were analyzed using ELISA kits for HBsAg and HBeAg (left panel). Cells were treated with lysis buffer for HBV core-associated DNA extraction, and HBV core-associated DNA was analyzed by real-time PCR (right panel). (**E**) Diagram of a mutant Sox4, in which the high-mobility group (HMG) of Sox4 was deleted. The mutant Sox4ΔHMG was sub-cloned into pCMV-Tag2A to generate pFLAG-Sox4ΔHMG. (**F**) HepG2 cells were co-transfected with pFLAG-Sox4 or pFLAG-Sox4ΔHMG and pHBV(wt). ChIP assays were performed to detect that Sox4ΔHMG can not bind to HBV DNA. (**G**) HepG2 cells were co-transfected with pFLAG-Sox4 or pFLAG-Soc4ΔHMG and pHBV1.3 (wt). HBsAg and HBeAg (left panel) and HBV core-associated DNA (right panel) were detected for indicating HBV replication level. *P < 0.05.

We then determined whether the specific binding of Sox4 protein to HBV DNA is necessary for HBV replication by using two approaches. First, two point mutations were introduced into the genome of HBV(wt) to change the sequence from AACAAAACAAAG to AATAAAACCAAG, generating a mutant HBV(mut) without altering the amino acid sequence of HBV HBp (Fig. 7A). We confirmed that the two point mutations of HBV(mut) had no effect on HBV protein expression and viral replication compared with HBV(wt) (Fig. 7B). Notably, ChIP assay showed that Sox4 could bind to HBV(wt) DNA (Fig. 7C, left panel), but failed to bind to HBV(mut) DNA (Fig. 7C, right panel), suggesting that the sequence AACAAAG of HBV genome is essential for the binding of Sox4 to the viral DNA. In addition, HBs and HBe expression (Fig. 7D, left panel) and HBV core-associated DNA replication (Fig. 7D, right panel) were activated by Sox4 in HepG2 cells transfected with HBV(wt), but not activated by Sox4 in the cells transfected with HBV(mut). These results demonstrated that the point mutation in Sox4 binding sequence of HBV genome abolishes the ability of Sox4 to activate HBV replication. The data also confirmed that the binding of Sox4 to HBV genomic DNA is sequence specific.

Second, a deletion mutant of Sox4 was constructed, in which the DNA binding domain, a high mobility group (HMG) domain, of Sox4 was deleted to generate a mutant Sox4ΔHMG (Fig. 7E). ChIP assay revealed that the wild-type Sox4 could bind to HBV(wt) DNA (Fig. 7F, left panel), but the mutant Sox4ΔHMG failed to bind to HBV(wt) DNA (Fig. 7F, right panel), suggesting that the HMG domain is essential for the binding of Sox4 to HBV DNA. In addition, HBs and HBe proteins expression (Fig. 7G, left panel) and HBV core-associated DNA replication (Fig. 7G, right panel) were activated by Sox4, but not by Sox4ΔHMG. These results demonstrated that the binding of Sox4 is essential for activating HBV replication, and Sox4 activates HBV replication by binding directly to the viral genomic DNA through its HMG domain.

## Discussion

**Figure 8:**
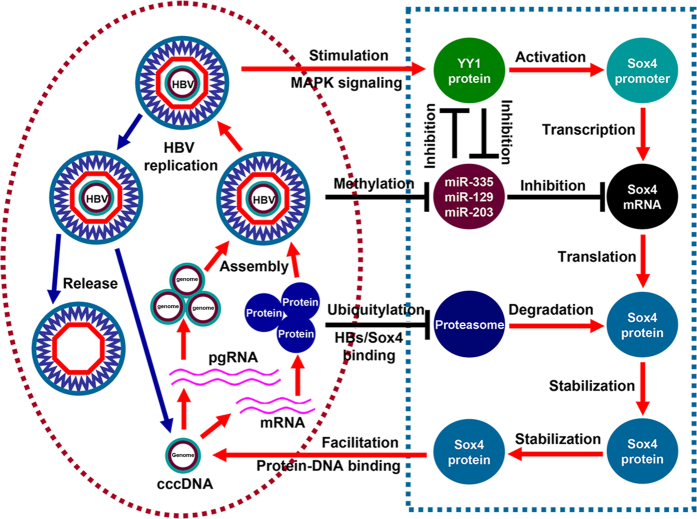
A proposed mechanism underlying the regulation of Sox4 expression and HBV replication. Sox4 expression is tightly controlled by HBV at multiple levels. HBV stimulates YY1 expression through the MAPK signaling, and YY1 subsequently enhances Sox4 promoter activity to activate Sox4 transcription. miR-335, miR-129-2 and miR-203 down-regulate Sox4 mRNA, whereas HBV suppresses the microRNAs expression via epigenetic modification to up-regulate Sox4 post-transcriptionally. Sox4 protein is degraded by polyubiquitin-dependent proteasome, while HBsAg protects Sox4 from degradation by interacting directly with Sox4, leading to up-regulating Sox4 post-translationally. Moreover, HBV-activated Sox4 facilitates the viral replication by binding directly to the viral genomic DNA via its HMG domain. Thus, HBV replication and Sox4 expression are highly correlated and tightly controlled by a positive feedback mechanism.

Chronic HBV infection is the result of a complex interaction between the replicating virus and the infected host cells that leads to progressive liver damage and increase the risk of developing liver cirrhosis and HCC^6^,^34^. However, the detailed mechanisms underlying the control of HBV replication, infection, and pathogenesis are largely unknown. In this study, we reveal a novel positive feedback mechanism by which HBV replication and Sox4 expression are closely related and tightly controlled (Fig. 8). Sox4 expression was previously reported to be regulated in many tissues and associated with cell transformation^35^, survival^36^, and metastasis^37^. However, the correlation between Sox4 expression and virus replication has not been reported. Here, we demonstrate that Sox4 expression is elevated in HBV-associated HCC tissues and HBV-transfected cells and that Sox4 expression is tightly controlled by HBV (Fig. 1), which provides the first evidence suggesting a correlation between Sox4 expression and viral infection. The mechanism by which HBV activates Sox4 expression was extensively elucidated. Further, we reveal that HBV stimulates Sox4 expression at multiple levels.

First, HBV activates Sox4 expression at the transcriptional level by inducing transcription factor YY1 expression through stimulating the MAPK signaling (Fig. 2). YY1 is a multifunctional transcription factor with fundamental roles in biological and physiological processes^38^. Increasing evidences suggest that YY1 plays important roles in tumorigenesis by regulating gene expression and acting as an activator, repressor, or initiator depending upon the cell types^39^. We demonstrate that YY1 is required for up-regulating Sox4 expression mediated by HBV and thus, reveal a new role of YY1 in activating Sox4 expression and an association between YY1 expression and viral infection.

In addition, HBV also activates Sox4 expression at the post-transcriptional level by inhibiting the expression of miR-335, miR-129-2, and miR-203 through epigenetic modifications (Fig. 3). Restoring miR-335 expression was reported to suppress lung and bone metastasis by interfering with Sox4 expression^40^. The miR-335 regulates cell proliferation, migration, and differentiation in human mesenchymal stem cells^41^ and activates p53 tumor suppressor pathway to limit cell proliferation and transformation^42^. The miR-129-2 inhibits cell proliferation by targeting CDK6^43^. In addition, miR-129-2 and miR-335 are suppressed in human pancreatic ductal adenocarcinoma (PDAC) and the miR-129-2/miR-335/Sox4/semaphorin-plexin regulatory axis plays a role in tumorigenesis^44^. The miR-203 is involved in regulating cell proliferation, migration, and invasion^45^,^46^,^47^, suppresses human malignancies including esophageal cancer and lung cancer^48^,^49^, and plays a pivotal role in melanosome maturation^50^. Here, we discover novel roles of miR-335, miR-129-2, and miR-203 in HBV-regulated Sox4 expression, and identify an association between viral infection and miR-335, miR-129-2, and miR-203 expression. Since HBV activates Sox4 expression not only through stimulating YY1 expression, but also by repressing miR-335, miR-129-2 and miR-203 expression, we thus verify the correlation between the two events. Interestingly, the miRNAs down-regulate YY1 expression by binding to its 3’-UTR, while YY1 suppresses the miRNAs expression (Fig. S2). Thus, we suggested that the two events are independent, but related.

Moreover, HBV enhances Sox4 expression at the post-translational level by protecting Sox4 from degradation through a direct interaction between the viral protein HBsAg and Sox4 protein (Fig. 4). Two main protein degradation pathways, the autophagy-lysosome pathway and the ubiquitin-proteasome pathway, exist in the cells^51^. We demonstrate that HBsAg directly interacts with Sox4 in the cytoplasm to protect Sox4 from degradation mediated by the ubiquitin-proteasome pathway (Fig. 4). This is consistent with a previous report showing that the C-terminal domain of Sox4 was demonstrated to regulate its polyubiquitin-independent proteasome degradation^32^. However, this study provides the first evidence demonstrating that the viral protein HBsAg protects the cellular protein Sox4 from proteasome-mediated degradation.

More interestingly, we reveal that the viral-activated Sox4 in turn, facilitates HBV replication by stimulating the virus protein expression and DNA replication in hepatoma cells (Fig. 5). Sox4 is a transcription factor with an important role in the embryonic development of heart^52^,^53^, pancreas^18^,^54^, and brain^55^. Sox4 also plays a central role in the epithelial-mesenchymal transition (EMT) and in primary tumor growth and metastasis^56^. In addition, Sox4 regulates many important cellular proteins, transcriptional regulators, and components of the RNAi machinery^57^. Thus, Sox4 is a crucial transcription factor that regulates many cellular functions. However, the role of Sox4 in viral replication has not been reported previously. This study at the first time demonstrates that Sox4 plays an important role in regulating viral replication.

In revealing the mechanism by which Sox4 activates HBV replication, we demonstrate that Sox4 activates HBV replication by directly binding to the viral genomic DNA through its HMG domain (Fig. 6 and Fig. 7). The HMG box of Sox4 binds preferentially to the AACAAAG motif in target genes^14^,^58^. Sequence analyses predicted that HBV genome contains a putative Sox4 binding site (AACAAAG) located at 884-855 nt in the genomic DNA, and interestingly, Sox4 recognizes this binding sequence to activate HBV replication. A mutation in the Sox4 binding site results in abolishing Sox4-mediated HBV replication, and a HMG deletion of Sox4 leads to the failure of Sox4 binding to HBV DNA. Thus, we provide the direct evidence to support that Sox4 activates HBV replication by binding to the viral genome.

In conclusion, HBV replication and Sox4 expression are closely correlated and tightly controlled by a positive feedback mechanism: HBV activates Sox4 expression at transcriptional, post-transcriptional, and post-translational levels through multiple mechanisms; the viral-activated Sox4, in turn, enhances HBV replication by binding directly to the viral genome (Fig. 8). Thus, we reveal a novel mechanism underlying Sox4 expression and HBV replication, identify a new role of Sox4 in regulating viral replication, and provide new insights into the mechanism underlying chronic HBV infection that leads to the development of liver diseases.

## Methods

### Clinical samples

The methods were carried out in accordance with the 1975 Declaration of Helsinki guidelines. Written informed consent was obtained from all subjects before clinic samples were collected. All experimental protocols were approved by the Ethics Committee of the College of Life Sciences, Wuhan University, China. Eight HBV-associated HCC tissues and the corresponding adjacent normal liver tissues were collected from Zhongnan Hospital of Wuhan University, China. All HCC tissues were confirmed positive for HBV and negative for hepatitis C virus (HCV), hepatitis D virus (HDV), and human immunodeficiency virus (HIV). The patients were not suffering from any concomitant illnesses and did not express any serological markers suggestive of autoimmune disease.

### Cell culture and transfection

The human hepatoma cell lines HepG2, Hep3B, HepG2.2.15, and Huh7, the normal liver cell line L02, and the human embryonic kidney cell line 293T (HEK293T) were purchased from the American Type Culture Collection (ATCC, Manassas, VA, USA) and cultured in Dulbecco’s modified Eagle medium (DMEM) (Gibco, Grand Island, NY, USA) supplemented with 10% heat-inactivated fetal bovine serum (FBS; Gibco) at 37 °C in a humidified atmosphere of 5% CO_2._ All cell lines were transfected with plasmids using Lipofectamine 2000 (Invitrogen, Carlsbad, CA, USA) following the manufacturer’s instructions.

### RNA extraction and real-time PCR

Total RNA was extracted from cells or transfected cells using TRIzol reagent according to the protocol provided by the manufacturer (Invitrogen). DNA was removed from the sample using on-column DNase I treatment. RNA was used as a template to synthesize cDNA using random primers and MMLV-RT (Promega, Madison, WI, USA) at 42 °C for 60 min, which was then denatured for 10 min at 70 °C. RT-PCR was performed using SYBR Green PCR master mix in a Light Cycler 480 (Roche Diagnostics Ltd., Risch-Rotkreuz, Switzerland). After an initial incubation at 95 °C for 5 min, the reaction mixtures were subjected to 40 cycles of amplification under the following conditions: 94 °C for 15 s, 56 °C for 15 s, and 72 °C for 20 s. The fluorescence was measured at this step to assess the quality of the primers, which was followed by a final melting curve step from 50 to 95 °C. The primers used in this study are listed in Table S3. Each sample was run in triplicate, and the threshold cycles (CT) were averaged and normalized to endogenous glyceraldehyde 3-phosphate dehydrogenase (GAPDH). The relative amount of amplified product was calculated using the comparative CT method. To determine the levels of core-associated DNA, RT-PCR was performed using a Light Cycler 480 real-time PCR instrument with a set of TaqMan real-time PCR primers that contained the 5′ primer P1, the 3′ primer P2, and the probe P3 (Table S3). The PCR reaction was performed as follows: 50 °C for 2 min, 95 °C for 10 min, and 40 cycles at 95 °C for 15 s and 60 °C for 60 s. The plasmid pHBV1.3 was diluted over a range of 10^7^-10^0^ and was used as a standard. All samples were analyzed in triplicate.

### HBV core-associated DNA extraction

HepG2.2.15 cells or HepG2 cells transfected with pBlue-HBV1.3 were lysed and centrifuged at 25 °C. Magnesium chloride was added to the supernatant. DNA not protected by HBV core protein was digested with deoxyribonuclease (DNase I). And then the lysates were treated with proteinase-K for 5 h at 55 °C. After phenol/chloroform extraction, HBV core-associated DNA was recovered by ethanol precipitation, and quantified by real time-PCR (RT-PCR) described above.

### Co-immunoprecipitation (co-IP)

HEK293T cells were seeded in 10-cm-diameter dishes and co-transfected with the indicated plasmids for 48 h, and then lysed in RIPA lysis buffer. Whole cell extracts were centrifuged at 10,000 × *g* for 5 min at 4 °C to remove cell debris. The supernatants were collected, pre-cleared using protein G Sepharose beads (GE Healthcare, Milwaukee, WI, USA), incubated with the indicated antibodies overnight at 4 °C, and then mixed with protein G sepharose beads (GE Healthcare) for 2 h at 4 °C. The immunoprecipitates were washed five times with RIPA lysis buffer, eluted with 1% SDS, boiled in loading buffer, and then analyzed using SDS-PAGE and Western blotting.

### Chromatin immunoprecipitation assay (ChIP)

ChIP assay was performed according to X-CHIP Protocol (Abcam, Cambridge, UK). Formaldehyde was added to culture medium to a final concentration of 1%. The cells were washed twice with PBS, scraped, and lysed in lysis buffer. Lysates were sonicated on ice and the debris was removed by centrifugation. One-fourth of the supernatant was used as DNA input. The remaining supernatant was diluted 10-fold with dilution buffer and incubated with the indicated antibody in the figures. Immunoprecipitated complexes were collected using protein G sepharose beads. The pellets were washed with dialysis buffer and incubated at 67 ^o^C for 5 h to reverse formaldehyde crosslink. DNA was precipitated with ethanol and extracted three times with phenol/chloroform. Pellets were re-suspended in TE buffer and subjected to PCR amplification using the corresponding primers.

### Protein degradation and ubiquitination assays

Cells were co-transfected with indicated plasmids and treated with the protein synthesis inhibitor CHX at a final concentration of 50 μg/ml for indicated times before harvesting. Proteins extracts were then prepared as described above and used for Western blotting. For ubiquitination assays, HEK293T cells in 10-cm-diameter dishes were co-transfected with the indicated plasmids for 36 h and then treated with the proteasome inhibitor MG-132 at a final concentration of 20 μM for 9 h. Cells were lysed in RIPA buffer and sonicated gently three times. Cell lysates were divided into two aliquots: one aliquot (5%) was used for Western blotting, and the other (95%) was incubated with anti-Sox4 and protein G Sepharose beads for 3 h at room temperature. The beads were washed five times using RIPA buffer, and the bound proteins were then eluted in 1% SDS sample buffer and analyzed by Western blotting.

### Statistical analyses

All experiments were reproducible and were repeated at least three times with similar results. Parallel samples were analyzed for normal distribution using Kolmogorov-Smirnov tests. Abnormal values were eliminated using a follow-up Grubbs test. Levene’s test for equality of variances was performed, which provided information for Student’s *t*-tests to distinguish the equality of means. Means were illustrated using histograms with error bars representing ± the standard deviation (SD); a *P* value of <0.05 was considered to be statistically significant.

## Author Contributions

J.S., Y.Z., X.G., J.M., X.X., Y.X., Y.L., K.W., and J.W. performed and analyzed the experiments. J.S., K.W., and J.W., designed the experiments. J.S., Y.Z., and J.W., wrote the manuscript.

## Additional Information

**How to cite this article**: Shang, J. *et al*. Hepatitis B virus replication and sex-determining region Y box 4 production are tightly controlled by a novel positive feedback mechanism. *Sci. Rep.* **5**, 10066; doi: 10.1038/srep10066 (2015).
